# Regulation of gut luminal serotonin by commensal microbiota in mice

**DOI:** 10.1371/journal.pone.0180745

**Published:** 2017-07-06

**Authors:** Tomokazu Hata, Yasunari Asano, Kazufumi Yoshihara, Tae Kimura-Todani, Noriyuki Miyata, Xue-Ting Zhang, Shu Takakura, Yuji Aiba, Yasuhiro Koga, Nobuyuki Sudo

**Affiliations:** 1 Department of Psychosomatic Medicine, Graduate School of Medical Sciences, Kyushu University, Fukuoka, Japan; 2 Department of Infectious Diseases, Tokai University of Medicine, Isehara, Japan; University of California Los Angeles, UNITED STATES

## Abstract

Gut lumen serotonin (5-hydroxytryptamine: 5-HT) contributes to several gastrointestinal functions such as peristaltic reflexes. 5-HT is released from enterochromaffin (EC) cells in response to a number of stimuli, including signals from the gut microbiota. However, the specific mechanism by which the gut microbiota regulates 5-HT levels in the gut lumen has not yet been clarified. Our previous work with gnotobiotic mice showed that free catecholamines can be produced by the deconjugation of conjugated catecholamines; hence, we speculated that deconjugation by bacterial enzymes may be one of the mechanisms whereby gut microbes can produce free 5-HT in the gut lumen. In this study, we tested this hypothesis using germ-free (GF) mice and gnotobiotic mice recolonized with specific pathogen-free (SPF) fecal flora (EX-GF). The 5-HT levels in the lumens of the cecum and colon were significantly lower in the GF mice than in the EX-GF mice. Moreover, these levels were rapidly increased, within only 3 days after exposure to SPF microbiota. The majority of 5-HT was in an unconjugated, free form in the EX-GF mice, whereas approximately 50% of the 5-HT was found in the conjugated form in the GF mice. These results further support the current view that the gut microbiota plays a crucial role in promoting the production of biologically active, free 5-HT. The deconjugation of glucuronide-conjugated 5-HT by bacterial enzymes is likely one of the mechanisms contributing to free 5-HT production in the gut lumen.

## Introduction

Enterochromaffin (EC) cells are located within the gut epithelia and store the largest pool of serotonin (5-hydroxytryptamine: 5-HT) in the body [1–3]. Thus, a significant amount of 5-HT is released into the gut lumen in response to various stimuli [1, 4–7]. For example, EC cells release 5-HT into the lumen in response to mechanical stimuli on the side of the gut mucosa, which enhances peristaltic reflexes [5, 8, 9]. Similarly, nutrients (e.g., short-chain fatty acids [SCFAs] and glucose), as well as acids and bases have been reported to induce the release of 5-HT from EC cells [10–12].

The influence of the gut microbiota on various aspects of human health and diseases has become a hot topic in the field of medical research. Gut bacteria play a critical role in the postnatal development of the mammalian immune system [13, 14], as well as in the development and functions of the central nervous system [15–18]. In fact, our previous studies using gnotobiotic mice demonstrated the importance of commensal microbiota in modulating the hypothalamic-pituitary-adrenal response to various stressors [19] and in shaping behavioral phenotypes of the host [20].

Reigstad and co-workers [21] reported that gut bacteria induce colonic 5-HT production via interaction with the SCFAs of EC cells. Indeed, the SCFAs acetate and butyrate were shown to induce tryptophan hydroxylase 1 (*TPH1*) mRNA expression in BON cells, a human EC cell line. Furthermore, Yano and colleagues [22] recently conducted an elegant study, which clearly demonstrated that gut microbiota can increase 5-HT synthesis by upregulating *TPH1* expression in EC cells in response to various biochemical substances of bacterial origin.

Although it is clear that the majority of 5-HT in the gut lumen is controlled by its release from the pool in EC cells in response to various stimuli, other mechanisms involving the gut microbiota are likely in play. Our previous studies showed that the bacterial β-glucuronidase enzyme of gut microbiota deconjugates catecholamines to generate free catecholamines in the gut lumen [23]. In general, 5-HT partially metabolizes to the glucuronide-5-HT metabolite (5-HT-*O*-glucuronide) in the liver [24]; hence, we hypothesized that the deconjugation process by bacterial enzymes may also contribute to free luminal 5-HT production in the gut.

To evaluate this possibility, in the current study, we determined luminal 5-HT levels throughout the gastrointestinal tract, and examined the involvement of gut microbes in the regulation of luminal 5-HT using germ-free (GF) mice and gnotobiotic mice that were recolonized with the fecal flora from specific pathogen-free (SPF) mice (EX-GF mice).

## Materials and methods

### Animals

GF and gnotobiotic mice were kept and treated according to previously reported methods [13, 19, 20]. In brief, GF BALB/c mice were originally obtained from the Central Institute for Experimental Animals (Kawasaki, Japan) and maintained in our laboratory. These mice have been bred for more than 10 generations in Trexler-type flexible-film plastic isolators with sterile food (CL-2, CLEA Japan Inc., Tokyo, Japan) and water. To reconstitute the GF mice with SPF gut microbiota, the parent GF mice were orally given the feces of male SPF mice. Their offspring thus became colonized with SPF microbiota at the neonate stage, and were used as the EX-GF mice. In the experiments examining the effects of microbial colonization on luminal 5-HT concentrations, the GF mice were orally administered 0.5 ml of 100-fold dilution of fresh SPF mouse feces. The conventionalization was done 3 (P3), 7 (P7), or 21 (P21) days before the conventionalized mice reached 10 weeks of age. All mice were sacrificed at 10 weeks of age. Only male mice were used in every experiment.

All experiments were approved by the Ethics Committee for Animal Experiments of Kyushu Universities (Permit Number: 23-115-0). All efforts were made to minimize animal suffering.

### Measurement of luminal and tissue 5-HT levels by high-performance liquid chromatography (HPLC)

Samples for luminal and tissue 5-HT measurement were prepared according to our previously described methods [20, 23, 25]. In brief, the mice were sacrificed by cervical dislocation, and then parts of the ileum, cecum, and distal colon were prepared as samples. The tissue and lumen samples were weighed and placed in 1.5-mL tubes. The samples were suspended in 1 mL of 0.01 M phosphate-buffered saline, and homogenized using homogenizers. After centrifugation for 15 min at 13,000 *g* at 4°C, the supernatants were collected and then mixed with 1 mL of 0.2 M perchloric acid (Sigma) for deproteinization. After centrifugation at 13,000 *g* for 15 min, the supernatant was adjusted to approximately pH 3.0 with 1 M sodium acetate. The resultant supernatant was filtered through a 0.2-μm filter (Millipore, Bedford, MA, USA), and 30 μL of the filtrate was applied to an HPLC system (Eicom, Kyoto, Japan) with a 150 mm × 3.0 mm octadecyl silane column (SC-5ODS; Eicom) and an electrochemical detector (ECD-300, Eicom) at an applied potential of +0.75 V versus an Ag/AgCl reference analytical electrode. Changes in the electric current (nA) were recorded on a computer using an interface system (Power Chrom ver 2.3.2.j, AD Instruments, Tokyo, Japan). The mobile phase contained 0.1 M aceto-citric acid buffer (pH 3.5), methanol, 0.46 M sodium 1-octane sulfonate, and 0.015 mM disodium ethylenediaminetetraacetic acid (830:170:1.9:1) at a flow rate of 0.5 mL/min. The 5-HT luminal and tissue concentrations were determined. The limit of detection of the system for all monoamines was 0.1 pg/sample.

### Measurement of conjugated 5-HT levels

The levels of glucuronide- and sulfate-conjugated 5-HT in the gut lumen were analyzed according to our previously described method for catecholamine measurements [23, 26, 27], with some modifications. We divided the deproteinized samples into three aliquots. All aliquots were adjusted to the indicated value of pH for the enzymatic reaction and incubated for 1 h at 37°C. The first aliquot (pH 7.0) was kept with no enzymes and used for free 5-HT measurement. The second aliquot was kept with β-glucuronidase (GUS, type IX from *Escherichia coli*, 750 units/sample; Sigma, St. Louis, MO, USA) for glucuronide-conjugated 5-HT measurement. The third aliquot was kept with sulfatase (type VI from *Aerobacter aerogenes*, 0.26 units/sample; Sigma). Enzymatic reactions were stopped by the addition of 1 mL of 0.2 M perchloric acid. After centrifugation, the supernatants were subjected to analysis on the HPLC system. The concentration of glucuronide-conjugated 5-HT was determined by calculating the difference between the first (free 5-HT) and the second (free 5-HT + glucuronide-conjugated 5-HT) value. Similarly, the concentration of sulfate-conjugated 5-HT was calculated as the difference between the first (free 5-HT) and the third (free 5-HT + sulfate-conjugated 5-HT) value.

### Reverse transcription-quantitative polymerase chain reaction (RT-qPCR) for 5-HT-related genes

The expression levels of 5-HT-related genes were analyzed using RT-qPCR according to the method described by Reigstad et al. [21]. We divided the colonic tissue into two fragments, the submucous-mucous layer and smooth muscle layer, and extracted total RNA from each fragment using a tissue homogenizer and the PureLink RNA Mini Kit (Ambion, USA). The smooth muscle layer contains the myenteric plexus. The extracted total RNA was then used to synthesize a random hexamer-primed cDNA template by reverse transcription with the SuperScript VILO Master Mix (Invitrogen, Carlsbad, CA, USA). Each 20-μL RT-qPCR consisted of 2× SYBR Premix EX Taq II (Tli RNaseH Plus, Takara Bio Inc., Japan) and 10 μM of gene-specific primers, according to the manufacturer protocols. Each sample was assessed in duplicates using the 7500 Real-Time PCR System (Applied Biosystems, Foster City, CA, USA), and mRNA expression levels were evaluated after normalization to beta-actin (*Actb*) mRNA expression using the ΔΔCT analysis method. All primer sequences used in this study are as follows: 5-hydroxytryptamine receptor 3A (*Htr3a)* forward, 5' -TGACCGCCTGTAGCCTTGAC-3', and reverse, 5'-TCCCACTCGCCCTGATTTATG-3'; 5-hydroxytryptamine receptor 4 (*Htr4)* forward, 5'-TGGAACAACATCGGCATAGTTGA-3', and reverse, 5'-CATAGGGCTTGTTGACCATGAAGA-3'; tryptophan hydroxylase 1 (*Tph-1)* forward, 5'-ACTGCGACATCAGCCGAGAA-3', and reverse, 5'- CGCAGAAGTCCAGGTCAGAAATC-3'; solute carrier family 6 member 4 (*Slc6a4)* forward, 5'- TTCGCCCAGGACAACATCAC-3', and reverse, 5'-TGACTGATGGATCTGCAGGACA-3'; *Actb* forward, 5'-CATCCGTAAAGACCTCTATGCCAAC-3', and reverse, 5'-ATGGAGCCACCGATCCACA-3'.

### Statistical analysis

All data are shown as the means ± SD. Changes between two groups were evaluated using Student’s t-test. Changes among three or more groups were evaluated by Dunnett’s post-hoc test after analysis of variance. All analyses were performed using the JMP Pro v.12.2.0 software package for Windows (SAS Institute Japan).

## Results

### Considerable amounts of 5-HT exist in the lumen of the gastrointestinal tract

**Fig 1:**
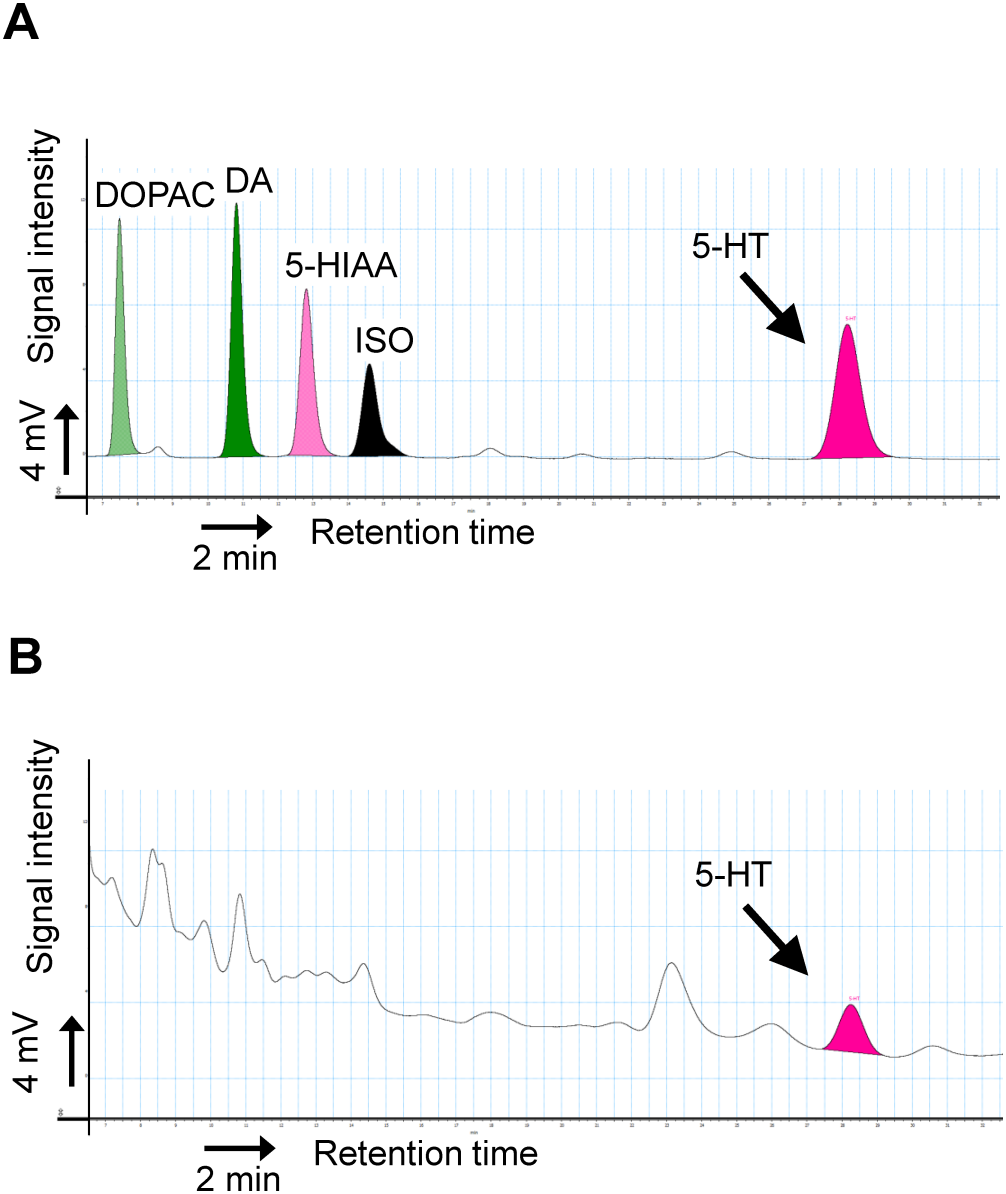
Luminal 5-HT concentrations in the colons of EX-GF mice. (A) Standard peaks of 3,4-dihydroxy-phenylacetic acid (DOPAC), dopamine (DA), 5-hydroxyindole acetic acid (5-HIAA), isoproterenol (ISO) and 5-HT in 0.02 M acetate buffer including 10 μM EDTA-2Na. (B) Representative chromatogram of the luminal contents of the colon. The 5-HT peak is indicated by arrows.

Fig 1 shows representative HPLC profiles of the 5-HT measurements in the colonic lumen. The presence of substantial contaminants inhibited the precise evaluation of luminal catecholamine-related metabolite levels (panel A); however, luminal 5-HT could be clearly identified as a unique peak (panel B).

**Fig 2:**
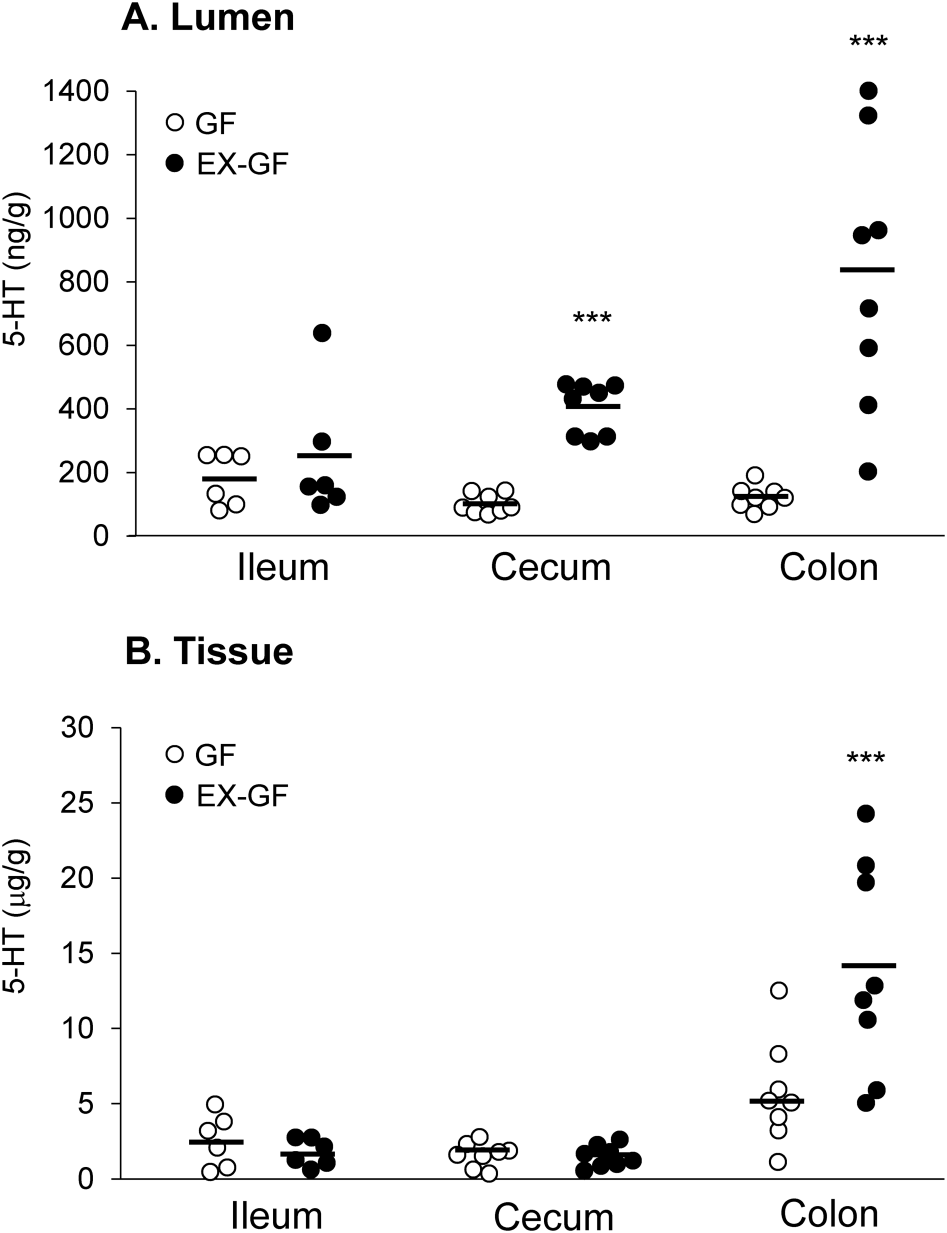
Luminal and tissue 5-HT concentrations in the gastrointestinal tract of germ-free (GF) mice and GF mice reconstituted with SPF mouse feces. (A) GF and EX-GF mice were sacrificed at 10 weeks of age. Luminal (n = 6–9) and (B) tissue (n = 6–8) 5-HT levels in GF (open circles) and EX-GF (closed circles) mice. *** P < 0.001 relative to the corresponding GF value.

As shown in Fig 2, 5-HT levels were higher in the EX-GF mice than in the GF mice in the lumens of the cecum and colon (P < 0.001). By contrast, there was no significant difference in ileal 5-HT levels between groups. For EX-GF mice, the luminal 5-HT level was highest in the colon among the parts of the gut. Tissue 5-HT levels in the colon were significantly higher in the EX-GF mice than in the GF mice (P < 0.05); however, there were no significant differences in either cecal or ileal tissue 5-HT levels between groups.

### Substantial elevation of 5-HT concentrations in the gut lumen after exposure to gut microbes

Various kinds of substances derived from the gut microbiota are known to have the ability to induce the release of 5-HT from EC cells [1, 24]. Therefore, to further clarify the involvement of gut microbes in the production of 5-HT in the gut lumen, we examined the time course changes in the 5-HT levels in the gut lumen of the GF mice upon exposure to the fecal microbiota of SPF mice.

**Fig 3:**
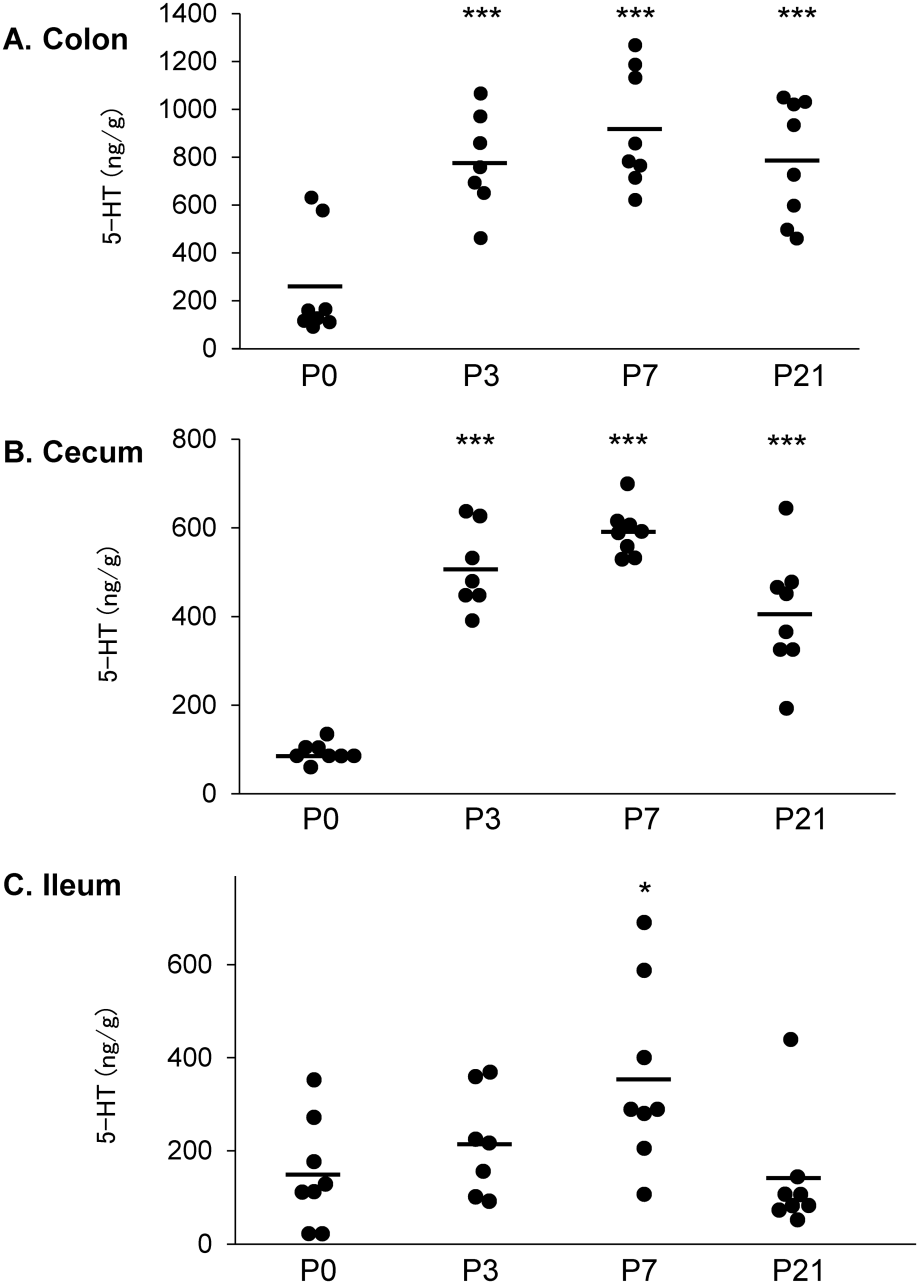
Dynamics of luminal 5-HT concentration in the gastrointestinal tract of GF mice after colonization with SPF feces. Male GF mice were sacrificed at 10 weeks of age for 5-HT measurements 3 (P3), 7 (P7), or 21 (P21) days after receiving SPF feces. GF mice without conventionalization were used as a control (P0). ***P < 0.001 and *P < 0.05 compared to the corresponding GF value.

As shown in Fig 3, the association with SPF microbiota rapidly induced a drastic elevation in luminal 5-HT levels in the cecum (Fig 3A) and colon (Fig 3B). A small and transient increase in 5-HT levels was found in the ileal lumen 7 days after the GF mice were exposed to SPF feces. In contrast, there were no significant changes in the tissue 5-HT contents after fecal administration during the observation period (data not shown).

### Effect of gut microbiota on the kinetics of 5-HT-related genes in the colon

Next, we investigated the gene expression of 5-HT and related molecules in the colon.

As summarized in Table 1, the *Tph1* and *Slc6a4* expression levels in the submucous-mucous layer were significantly decreased on day 3 after exposure to SPF feces compared with the basal levels; however, they returned to basal levels at 21 days after exposure to the SPF feces. In the smooth muscle layer, the *5-Htr3a* mRNA levels were significantly increased at 21 days after SPF fecal inoculation in comparison to the basal levels. However, there were no significant changes in *5-Htr4* mRNA levels before and after SPF fecal administration.

**Table 1:** Relative expression levels of 5-HT-related genes determined with RT-qPCR.

	Basal level	P3	P21
*Htr3a*	1.36 ± 0.73	1.27 ± 0.37	2.06 ± 0.45*
*Htr4*	1.17 ± 0.26	1.39 ± 0.51	1.58 ± 0.56
*Tph1*	0.93 ± 0.19	0.45 ± 0.19*	0.95 ± 0.50
*Slc6a4*	0.81 ± 0.19	0.56 ± 0.17*	0.72 ± 0.16

Male GF and conventionalized mice were sacrificed for RT-qPCR analysis at 10 weeks of age. Colonic samples for RT-qPCR were obtained at 3 (P3) and 21 (P21) days after administration with feces from SPF mice. GF mice at 10 weeks of age were used as a control (basal level).

*, significant difference (P < 0.05) from the corresponding GF level. *Htr3a*, 5-hydroxytryptamine receptor 3A; *Htr4*, 5-hydroxytryptamine receptor 4; *Tph1*, tryptophan hydroxylase 1; *Slc6a4*, solute carrier family 6 member 4.

### Gut microbiota exposure increases the amount of unconjugated, free 5-HT in the gut lumen of GF mice

Our previous report [23] showed that a large proportion of catecholamines in the gut lumen of GF mice exists in a conjugated form that is biologically inactive. Therefore, to clarify the contribution of gut microbes to the production of free 5-HT levels in the gut lumen, both the free and conjugated forms were measured in the colons of GF mice, and compared with those in the EX-GF mice.

**Fig 4:**
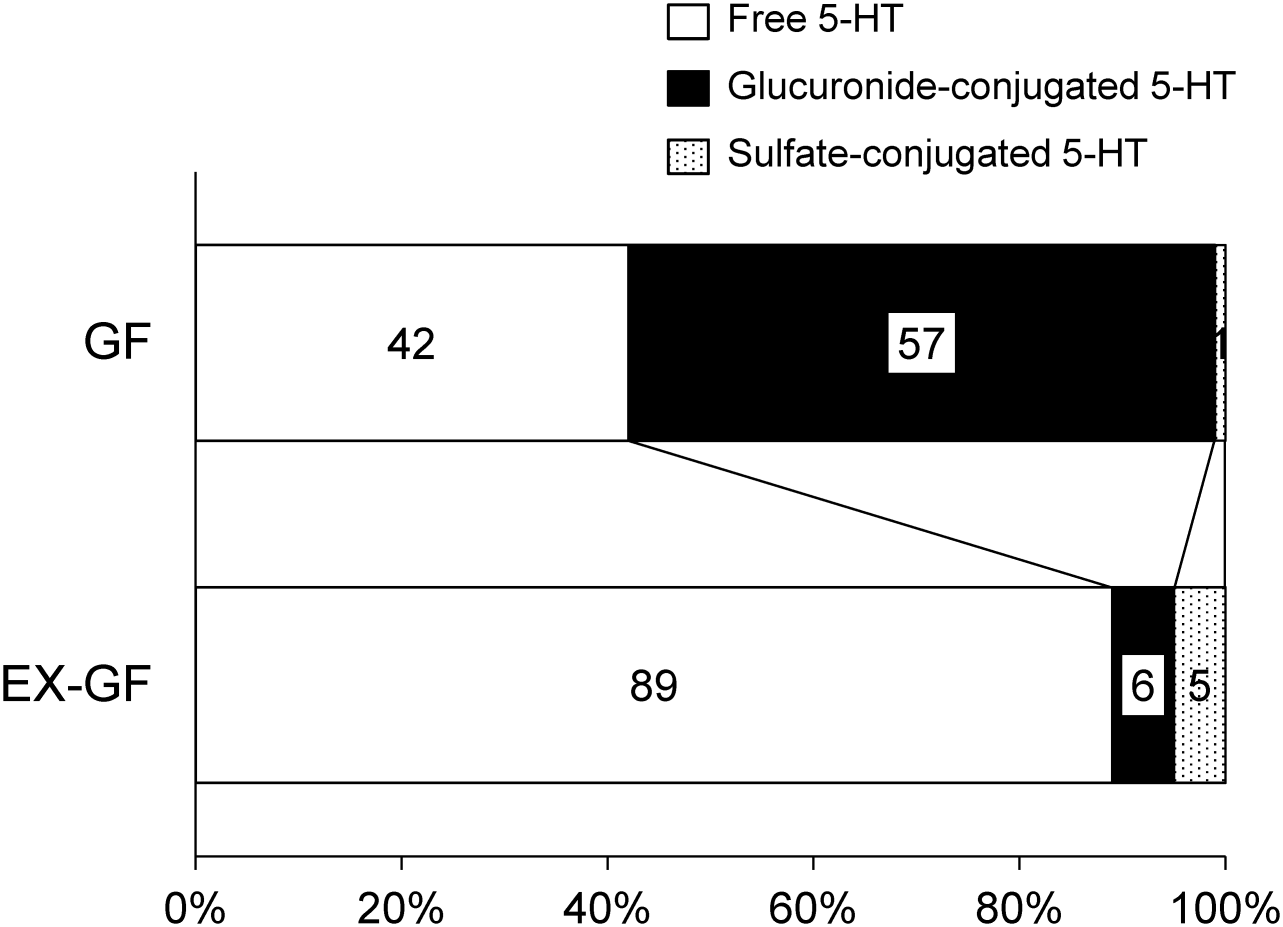
Free, glucuronide-conjugated, and sulfate-conjugated 5-HT levels in the colonic lumens of GF and EX-GF mice. GF and EX-GF mice were sacrificed at 10 weeks of age. Luminal free (open bars), glucuronide-conjugated (closed bars), and sulfate-conjugated (dotted bars) 5-HT levels in the colons of GF (n = 8) and EX-GF (n = 8) mice. The mean value of each form of 5-HT is expressed as the percentage of the total 5-HT (free 5-HT + conjugated 5-HT).

As shown in Fig 4 and Table 2, approximately 90% of the 5-HT was found in an unconjugated (free) form in the EX-GF mice, whereas, more than 50% of the 5-HT was found in a glucuronide-conjugated form in the GF mice. Interestingly, a significant amount of sulfate-conjugated 5-HT was only found in the colon of EX-GF mice. The total 5-HT levels in the colonic lumen of the EX-GF mice were twice as high as those of the GF mice. Taken together, these results indicate that gut microbes play a crucial role in promoting luminal 5-HT production, and that the deconjugation process of glucuronide-conjugated 5-HT by bacterial enzymes may contribute to the enhanced production of free 5-HT in the gut lumen.

**Table 2:** Levels of free and conjugated 5-HT in the colonic lumen of GF and EX-GF mice^a^.

	Total (ng/g)	Free (ng/g)	G-conjugated (ng/g)	S-conjugated (ng/g)
GF	252 ± 89.0	106 ± 33.0	143 ± 73.0	1.4 ± 1.9
EX-GF	563 ± 259*	501 ± 255**	32.1 ± 9.91**	29.4 ± 7.19***

^a^Free, glucuronide (G)-conjugated, and sulfate (S)-conjugated 5-HT levels were measured in the colonic lumens of GF and EX-GF mice (10 weeks of age, male, n = 8) as described in the Methods. Total 5-HT levels were calculated as the sum of free, G-conjugated, and S-conjugated 5-HT.

***P < 0.001,

**P < 0.01, and

*P < 0.05 compared to corresponding GF values.

## Discussion

Recent research via animal experiments and human studies suggests that the gut microbiota plays a crucial role in 5-HT synthesis and its regulation in the gut lumen. In support of this interaction, the present study showed that EX-GF mice, which were GF mice reconstituted with SPF microbiota, had higher levels of luminal 5-HT in the cecum and colon, as well as whole-colon 5-HT levels compared with the GF mice. These direct effects were confirmed with direct exposure experiments, in which 5-HT levels in the gut lumen of GF mice rapidly and markedly increased within 3 days after oral administration of SPF mouse feces. In contrast, the mRNA expression levels of *Tph1*, an important molecule involved in the 5-HT synthesis pathway, showed a transient decrease at 3 days after the exposure, and returned to the basal level 21 days later. The majority of 5-HT was in the free form in the EX-GF mice, whereas more than 50% of the 5-HT was in the glucuronide-conjugated form in the GF mice, indicating the critical role of gut microbes in the production of biologically active, free 5-HT.

Uribe and co-workers [28] reported that the microbiota influences the number of gut endocrine cells and the release of biologically active peptides based on comparisons between GF and conventional rats. Moreover, the results of a metabolome analysis [29] showed that the plasma 5-HT levels of GF mice were 2.8 times as high as those of conventionalized mice. More recently, Yano and co-workers [22] demonstrated that commensal microbes such as spore-forming bacteria induce 5-HT biosynthesis from EC cells in the colon, providing a supply of free 5-HT to the gut mucosa and lumen as well as the circulating platelets. The present results confirmed such a microbe-induced 5-HT production mechanism in the gut given that the tissue 5-HT levels were higher in the EX-GF mice than in the GF mice.

Besides mechanical stimuli, luminal glucose and SCFAs have been shown to release 5-HT from EC cells [21]. In addition, Braun and colleagues [30] reported that odorant substances found in the lumen of the gut can stimulate serotonin release via olfactory receptors in an EC cell line, BON cells. The rapid increase in luminal 5-HT levels upon exposure to SPF feces observed in the present study is consistent with the above findings, suggesting that biochemical substances derived from the gut microbiota can stimulate 5-HT release from EC cells, thus contributing to the total 5-HT pool in the gut lumen.

It should be noted that there was huge variation in the luminal and tissue levels of 5-HT in the colon of EX-GF mice. The reason for this variability is not currently clear: however, it may be related to a technical problem during colon tissue collecting. For example, mechanical stimuli to the mucosa can induce the release of 5-HT from EC cells [8, 9], which may have contributed to the large variation of luminal 5-HT measurement. Clearly, further studies are needed to clarify the reason for this variation observed.

In the current study, after exposure to SPF feces, a transient decrease in *Tph1* and *Slc6a4* mRNA expression levels was observed in the submucous-mucous layer of the colon. Interestingly, the expression levels of these gene were also reportedly reduced in the bowel of patients with irritable bowel syndrome [31, 32]. The precise mechanism whereby microbial colonization down-regulates these genes has not yet been elucidated: however, such a decrease may be an adaptive response to excessive 5-HT release after exposure to a microbial burden, and thus regulate the hypersensitivity of the 5-HT system in the gut. In contrast, *5-Htr3a* mRNA expression level in the muscle layer of the colon was increased upon exposure to gut microorganisms. Recently, Zeng and co-workers [33] showed that the *5-Htr3a* mRNA level in enteric neurons is increased through inflammation-induced glial cell-derived neurotrophic factor (GDNF). These data suggest that gut microbiota may up-regulate *5-Htr3a* expression via inducing GDNF on gut epithelial cells, and contribute to the increased sensitivities of enteric neurons to 5-HT. We do not have actual data supporting this speculation; hence, further studies are critically important for clarifying the interaction among gut microbes, GDNF and visceral hypersensitivities.

Conjugation by glucuronidation and sulfation is known to play an important role in the metabolism of many exogenous and endogenous compounds, which is mostly performed in the liver [34, 35]. Our previous study clearly demonstrated that glucuronide- and sulfate-conjugated catecholamines excreted in the gut via the bile duct are hydroxylated by bacterial β-glucuronidase in the gut lumen where they are found in a biologically active and free form [23]. 5-HT is degraded to 5-hydroxyindoleacetic acid by monoamine oxidase A at the gut epithelial level [36]. However, when 5-HT enters the portal circulation, it partially metabolizes to the glucuronide-5-HT metabolite (5-HT-*O*-glucuronide) in the liver [24]. This background combined with the current finding that the majority of 5-HT found in the EX-GF mice was in the free form suggest the following two mechanisms underlying the microbial regulation of luminal 5-HT levels: a large part of luminal 5-HT is released from EC cells in response to various stimuli, whereas a small but significant amount is produced by the deconjugation of 5-HT-*O*-glucuronide by a bacterial enzyme such as β-glucuronidase. Interestingly, a significant amount of sulfate-conjugated 5-HT was only found in the colon of EX-GF mice, indicating an indispensable role of gut microbes in the production of sulfate-conjugated 5-HT. The liver expression levels of sulfotransferases, a group of phase II enzymes that catalyze the sulfation of substrates, were reported to be higher in SPF mice than in GF mice [37, 38]. Therefore, increased amounts of sulfate-conjugated 5-HT in the colonic lumen may result from the increased sulfation of 5-HT by the microbe-induced up-regulation of sulfotransferases in the livers of EX-GF mice.

In conclusion, our results further support the current view that the gut microbiota plays a crucial role in generating 5-HT synthesis and regulation in the gut. The deconjugation process of glucuronide-conjugated 5-HT is considered to be one of the mechanisms whereby gut microbes can produce free 5-HT in the gut lumen.
